# Anatomy of the Cun Position at Wrist and Its Application in Pulse Diagnosis

**DOI:** 10.1155/2019/1796576

**Published:** 2019-05-07

**Authors:** Peng Li, Yi-kuan Du, Xiang-nan Chen, Su-ming Jiang, Jin-sheng Liu, Chun Yang, Xue-peng Zhang

**Affiliations:** ^1^Department of Human Anatomy, Shantou University Medical College, Shantou, Guangdong, 515041, China; ^2^Rehabilitation Department, Dongguan People's Hospital, Dongguan, Guangdong, 523059, China; ^3^Department of Traditional Chinese Medicine, The Second Affiliated Hospital of Shantou University Medical College, Shantou, Guangdong, 515041, China; ^4^Department of Human Anatomy, Guangdong Medical University, Dongguan, Guangdong, 523808, China; ^5^School of Zhang Zhongjing National Medicine, Nanyang Institute of Technology, Nanyang, Henan, 473004, China

## Abstract

Information on anatomy of the Cun position at wrist is lacking; whether the blood vessel taking pulse in Cun is the radial artery or the superficial palmar branch is also clinically controversial. The objective was to investigate the boundaries and contents, and the vascular distribution and their pulse points in Cun. Thirty-two upper extremities of 16 human cadavers were investigated for dissection and observation. The boundaries, contents, and blood vessel distribution in Cun were observed; the location of pulse points in Cun was identified; the length of the superficial palmar branch in wrist pulse (L1), the pulp width of the index finger (L2), and the angle between the radial artery and the superficial palmar branch were measured. The results showed that the Cun was located in the region formed by the bulge of the prominent bone proximal to the palm, the radial flexor tendon, the tubercle of scaphoid, and the abductor longus muscle tendon. In this area, the radial artery could be pulsed part in the medial side of the abductor longus muscle tendon, while the superficial palmar branch lied near the surface and was easy to pulse in the lateral side of the radial flexor tendon and the medial side of the tubercle of scaphoid. The ratio of L1 to L2 was 1.2±0.8, and the angle was 23.3±9.9°. The results suggested that it could not be generalized that the blood vessel taking pulse in Cun was the radial artery or the superficial palmar branch; it might depend on the vascular distribution in Cun, the region of finger positioning, and the patient's pulse condition.

## 1. Introduction

In traditional Chinese medicine, wrist pulse-taking method is an approach by which a practitioner understands the pathologic properties of a disease (including yin and yang, exterior and interior, cold and heat, deficiency and excess) and implements syndrome differentiation and treatment. It is one of the four examinations (inspection, listening and smelling examination, inquiry, and pulse taking and palpation) and plays a key role [1]. Scientific research using modern transducers and computer technology has been performed to detect the wrist pulse during the last thirty years [2]. For example, pulse analysis in thirty human subjects with bipolar disorder and thirty nonpsychotic human subjects showed that the harmonic values of Lung Meridian and the harmonic percentage of Spleen Meridian and Lung Meridian on the right hand were significantly different between the two groups [3]. Pulse pressure waveforms from radial artery at right Guan of 30 patients with dyspepsia were significantly greater than those of 30 normal subjects. This study suggested the pulse-frequency spectrum at right Guan was a more effective characteristic for dyspepsia patients [4]; however, pulse waveform harmonics based on the resonance theory showed that no marked differences were observed on the identified internal organ among the Chi-Guan-Cun positions. This study suggested that the depth of the radial artery but not the positions should be emphasized for pulse waveform analysis [5]. Although a lot of work has been done on the pulse diagnosis, these are far from enough to explain the rich connotation of the wrist pulse-taking method in traditional Chinese medicine, and there are still many problems to be further studied

Wrist pulse located at wrist is divided into three regions, namely, Cun, Guan, and Chi, which are used in pulse diagnosis to assess the health status of specific internal organs [5]. According to the systematic correspondence between organs and pulses, left Cun is used to detect the health status of the heart/small intestine and right Cun is used to detect the health status of the lung/large intestine in traditional Chinese medicine [1, 6]. In traditional medicine, the Guan position is first determined at the medial portion of the bulge of the prominent bone proximal to the palm, and then the Cun position is determined to be located adjacent and distal to the Guan position [1, 6]. It was generally believed that the blood vessel where the pulse was taken in Cun was the radial artery [1, 7]. However, Huayun Luo proposed that the radial artery could not be taken at traditional pulse position in Cun, but the pulse could be obviously taken when moving the finger towards the ulnar side, so he considered that the blood vessel taking pulse in Cun was the superficial palmar branch [8]. Because the blood vessel taking pulse is helpful to find the exact Cun position, it is needed to determine whether the blood vessel is the radial artery or the superficial palmar branch in Cun. Although some articles have discussed blood vessels from the anatomical point of view [8, 9], some articles have focused on the superficial palmar branch on flap transplantation [10–12], and some articles have discussed the existence of anatomical variations of arteries in the upper limb which could also impact the pulse diagnosis [1, 13–15], but the human cadavers study in Cun has been lacking.

The purpose of this study was to investigate the boundaries and the contents in Cun, as well as the distribution and pulse point of the radial artery and the superficial palmar branch, and to make this information available for use in pulse diagnosis.

## 2. Methods

### 2.1. Materials and Methods

This study was performed using 32 upper limb specimens of 16 human cadavers (eight male and eight female). The bodies were donated to the Division of Anatomy of Shantou University Medical College by people who had given their informed consent to use their bodies for scientific purposes prior to death. All cadavers were preserved using a formaldehyde-phenol solution.

Macroscopic measurements were performed with the forearm in lateral rotation position. Dissection and observation were done by the professional anatomy teachers and technicians. All measurements were performed by the first author (MD) using a digital slide caliper and a square ruler.

### 2.2. Dissection and Observation

The cadaver was placed in the supine position with the forearm rotated laterally and the palms facing forwards. Firstly, we observed and touched the surface structures at wrist, then we cut and removed the skin to trace the superficial veins and cutaneous nerves, then we incised the deep fascia and cleaned the radial vessel and its branches, and finally we examined the boundaries, the contents, and the vascular distribution in Cun [16].

We photographed anatomical structures of the Cun position at wrist. We measured the length of the superficial palmar branch in wrist pulse (L1) (Figure 3(c)) and the pulp width of the index finger (L2) (Figure 3(d)) and calculated their ratio. We also measured the angle between the radial artery and the superficial palmar branch (*α*) (Figure 3(c)).

### 2.3. Statistical Methods

We analyzed the data using SPSS 13.0 software (SPSS, Chicago, IL, USA). All data were presented as means ± standard deviations. The relationship between the length of the superficial palmar branch and the width of the index finger was evaluated by scatter plot and frequency distribution.

## 3. Results

### 3.1. Surface Anatomy and Superficial Dissections of the Cun Position at Wrist

**Figure 1:**
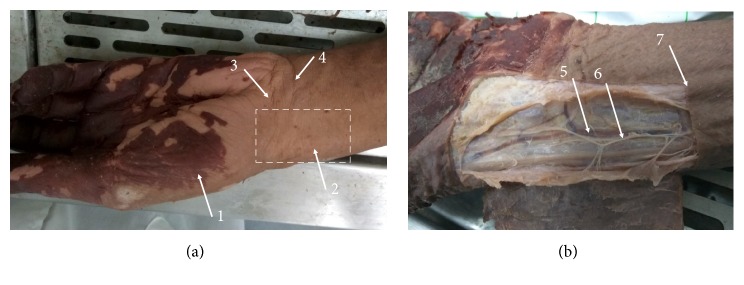
The left wrist and hand anterior (palmar) view. (a) Surface anatomy. (b) Superficial dissections. 1. Thenar eminence. 2. Bulge of the prominent bone proximal to the palm. 3. Distal wrist crease. 4. Middle wrist crease. 5. Tributaries of the cephalic vein. 6. Lateral antebrachial cutaneous nerve. 7. Flexor carpi radialis tendon.

According to the positions of the wrist pulse in traditional Chinese medicine, firstly we touched the bulge of the prominent bone proximal to the palm [6] and then placed the index finger, middle finger, and ring finger, respectively, in Cun, Guan, and Chi. We found that the Cun was located on the anterior area of the radiocarpal joint; its surface was roughly between the middle wrist crease and the distal wrist crease (Figure 1(a)). We also found the thenar eminence at the distal aspect of Cun and touched the flexor carpi radialis tendon at the medial (ulnar) aspect of Cun (Figure 1(b)).

When the skin was removed, we found the tributaries of the cephalic vein ascended along wrist pulse and the vein formed anastomoses with the radial venae in deep; the terminal branches of the lateral antebrachial cutaneous nerve and the superficial radial nerve were distributed separately in the medial and the lateral of the bulge of the prominent bone proximal to the palm (Figure 1(b)).

### 3.2. Deeper Dissections of the Cun Position at Wrist

**Figure 2:**
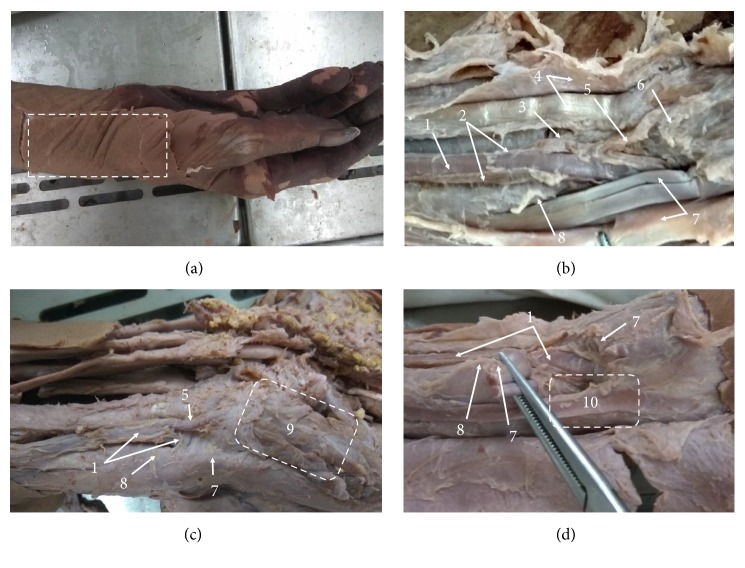
The right wrist and hand anterior (palmar) view and lateral (radial) view. (a) Anatomical area. (b) Deeper dissections. (c) The superficial palmar branch in Cun overflows to the thenar eminence in the palm of the hand. (d) The radial artery bent around the radial styloid process to the anatomical snuff box in the dorsal aspect at wrist. 1. Radial artery. 2. Radial venae. 3. Radial palmar carpal branch. 4. Flexor carpi radialis tendon and tendon sheath. 5. Superficial palmar branch. 6. Tubercle of scaphoid. 7. Abductor pollicis longus tendon and tendon sheath. 8. Bulge of the prominent bone proximal to the palm. 9. Thenar eminence 10. Anatomical snuffbox.

When the subcutaneous tissue and the deep fascia were removed, we found the Cun was located in a quadrilateral depression formed by bones and tendons at the anterior area of the radiocarpal joint; specifically, the proximal border was the bulge of the prominent bone proximal to the palm, the distal border was the tubercle of scaphoid, the medial (ulnar) border was the radial flexor tendon and tendon sheath, and the lateral (radial) border was the abductor longus muscle tendon, the extensor pollicis brevis tendon, and their tendon sheath (Figure 2(b)).

In Cun, we found one radial artery and two accompanying radial veins; at the proximal Cun, the radial artery descended along the medial side of the bulge of the prominent bone proximal to the palm and the abductor longus muscle tendon (Figure 2(b)). It could be pulsed part in this area. At the distal Cun, the radial artery was covered by thickened deep fascia (Figure 2(c)) and bent around the radial styloid process to the anatomical snuff box in the dorsal aspect at wrist when the tendon of the abductor longus muscle and the extensor pollicis brevis were cut and opened (Figure 2(d)); this position was deep and it was difficult to pulse the radial artery. When the radial vessels were pulled to the lateral, we found the radial palmar carpal branch sent from its posterior wall and was hidden inside (Figure 2(b)); we also found the superficial palmar branch arose from the radial artery, descended along the lateral side of the radial flexor tendon, and entered the thenar eminence on the medial side of the tubercle of scaphoid, and it lied near the surface and was easy to pulse (Figures 2(b) and 2(c)).

### 3.3. Measurement of the Cun Position at Wrist

**Figure 3:**
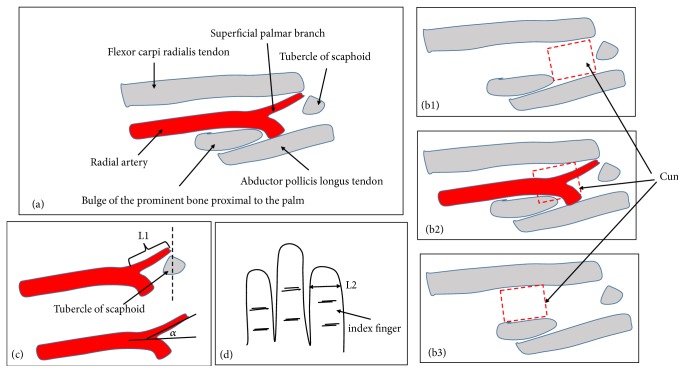
Cun position of the right wrist and measurement anterior (palmar) view. L1: the length of the superficial palmar branch in wrist pulse; L2: the pulp width of the index finger; *α*: the angle between the radial artery and the superficial palmar branch.

We described the mean values and standard deviations (SD) of the length of the superficial palmar branch in wrist pulse, the pulp width of the index finger, and their ratio as well as the angle where the radial artery sent the superficial palmar branch (Figure 3). These data were summarized in Table 1.

**Figure 4:**
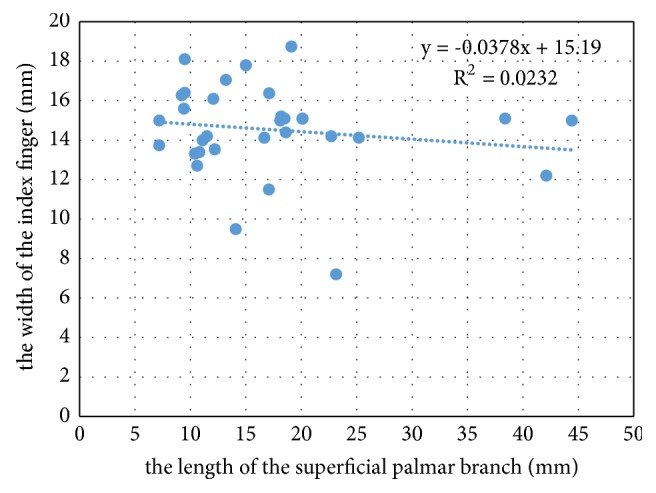
Scatter plot of the length of the superficial palmar branch versus the pulp width of the index finger of 32 upper limb specimens.

**Figure 5:**
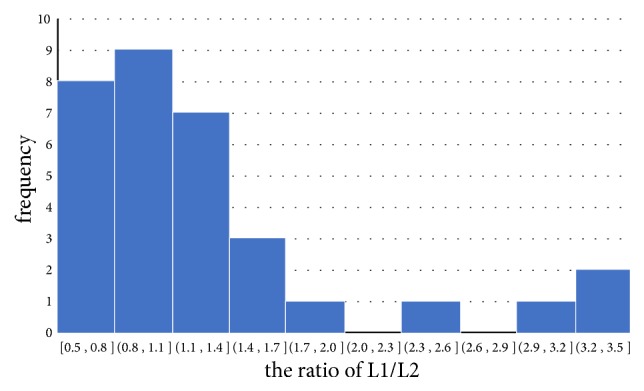
Frequency distribution of the ratio between the length of the superficial palmar branch (L1) and the pulp width of the index finger (L2).

We draw the scatter plot of the length of the superficial palmar branch versus the pulp width of the index finger (Figure 4). It was notable that the superficial palmar branch varied greatly at length between 25mm and 45mm, suggesting that we should notice the variation of the superficial palmar branch in pulse diagnosis. We have also drawn the frequency distribution of the ratio between the length of the superficial palmar branch and the pulp width of the index finger (Figure 5). It was notable that cases with a ratio greater than 0.8 accounted for the majority, suggesting that the pulse of the superficial palmar branch could be felt by the index finger.

## 4. Discussion

### 4.1. Boundaries and Contents in Cun

In traditional Chinese medicine, the description of three regions (Cun, Guan, and Chi) at wrist was relatively simple and significantly different from the description in human anatomy, especially in the orientation and directional terms. “*Binhumaixue*”, written by Shizhen Li in the Ming Dynasty, recorded that Guan lied in the bulge of the prominent bone proximal to the palm; yang was in front of it, and yin was behind it; Cun lied in yang, and Chi lied in yin [6]. The Chinese Medicine Diagnostics, written contemporarily, recorded that wrist pulse on each side of the hand was divided into three regions, namely, Cun, Guan, and Chi; marked by the styloid process of radius, its inner part was Guan, the front of Guan (wrist end) was Cun, and behind Guan (elbow end) was (is) Chi [1]. In modern anatomy, “the bulge of the prominent bone proximal to the palm” [6] observed by the ancient physicians refers to the radial tubercle at the proximal aspect of the wrist crease and the styloid process. The styloid process, in fact, is the projection of bone on the lateral aspect of the distal radius bone [1]. Some studies have pointed out that the two structures were different and should not be confused [7, 17], which coincides with our observation [18]. In addition, the “front” and “back” referred to by the ancients actually referred to distal (or wrist) and proximal (or elbow) in human anatomy.

To avoid ambiguity, this article used the anatomical position and the orientation and directional terms to describe the structure of the wrist. In this position, a person stood erect, with the upper limbs lying by the side and the palms facing forwards; structures in front of the body were anterior, whereas structures behind the body were posterior; structures nearer the head were superior, whereas structures closer to the feet were inferior; structures closer to the point of attachment of upper limb to the body trunk were proximal, whereas structures farther from the point of attachment of upper limb to the body trunk were distal; structures on the inner side of the upper limb were the medial (ulnar), whereas structures on the outer side were the lateral (radial) [18].

Although the location of Cun has been partially described in some studies, the detailed anatomical description of Cun is lacking. For example, a survey of 78 adult subjects has shown that the Cun was approximately 1.14 cm distal from Guan [7]. Jang-Han Bae defined the region of the Cun as that between the styloid process and the prominent bone [17], while Tyan C. C thought the location of Cun was from the distal wrist crease to the highest point of “prominent bone” [19].

According to the Cun positions at wrist in traditional Chinese medicine, this study described its boundaries and contents. This study showed that the Cun was located on the lateral side of the wrist, between the distal end of the radius and the scaphoid. The surface was roughly between the middle wrist crease and the distal wrist crease. It was deeply located in the region formed by the bulge of the prominent bone proximal to the palm, the radial flexor tendon, the tubercle of scaphoid, and the abductor longus muscle tendon (Figure 3(b1)). It contained the branches of the lateral cutaneous nerves of the forearm and the cephalic vein in superficial fascia, the radial vessel, and its branches in deep. We found that the typical feature of the Cun was located at the joint and its movement was large; those caused skin folds and blood vessel bending in this area. According to these characteristics and the fact that traditional practitioners put a pillow under the patient's wrist in pulse diagnosis, we think the purpose is to permit the wrist extension and keep the blood vessels straightened.

### 4.2. Vascular Distribution and Pulse Point in Cun

We observed and compared the distribution and pulse point felt with the index finger between the radial artery and the superficial palmar branch in Cun. In this area, the radial artery passed around the medial side of the bulge of the prominent bone proximal to the palm and the abductor longus muscle tendon and bent to the anatomical snuff box in the dorsal aspect at wrist; it could be pulsed part at proximal Cun but not at distal Cun. This distribution may explain the phenomenon of pulse loss at traditional pulse position in Cun [8]. In Cun, the superficial palmar branch arose from the radial artery, descended along the lateral side of the radial flexor tendon, and entered the thenar eminence on the medial side of the tubercle of scaphoid. It was located on the ulnar side of the radial artery, and the angle between them in this study was 23.3±9.9°. It lied near the surface and was easy to pulse; this was consistent with Huayun Luo's clinical report that “if the pressed position moves to the ulnar side about 0.5cm, the pulse in Cun could be felt clearly” [8]. In traditional Chinese medicine, a practitioner takes pulse in Cun, Guan, and Chi with pressing through the index finger, middle finger, and ring finger, respectively. Therefore, the blood vessels of the Cun can be felt by the index finger, and the length of the blood vessel of the Cun should be at least close to the pulp width of the index finger. In the study, we measured the length of the superficial palmar branch and it was about 17.3 mm, the pulp width of the index finger was about 14.5 mm, and the ratio between them was 1.2±0.8; these indicate that most of the superficial palmar branches can be felt by the index finger and may be used as a clue to find the Cun position. In addition, if pressure is applied for pulse detection, the index finger contact surface will become wider and L2 will become larger than these measured in this study.

Many ancient medical books recorded that the pulse in Cun overflowed to the thenar eminence, and this was consistent with the characteristics that the superficial palmar branch passed through the thenar eminence into the palm of the hand. For example, the Long Pulse, recorded in “*Mailiqiuzhen”*, was felt lengthily, up to the thenar eminence, down to Chize point, more than the length in Cun, Guan, or Chi, regardless of shallow or deep [20]. The clinical case about Hematemesis in* “Linzhengzhinanyian”* recorded that the right pulse was long, big, and up to the thenar eminence [21]. The article in “*Yangkegangyao”* recorded that when qi and fire in the upper energizer were boiling, the pulse must reflect the state and overflow, even beyond the Cun and up to the thenar eminence [22]. Those articles suggested that the pulse felt among many diseases in the upper energizer disease syndrome, excess fire syndrome, and syndrome of yin deficiency with yang hyperactivity should be the superficial palmar branch; however, we should pay attention to the fact that the classics did not mention whether pulse changes related to the underlying syndrome were permanent or temporary.

According to the distribution characteristics of the blood vessels, this study discussed the location of pulse points in Cun. The pulse point of the radial artery should be taken in the medial side of the bulge of the prominent bone proximal to the palm and the abductor longus muscle tendon, while the pulse point of the superficial palmar branch should be taken in the lateral side of the radial flexor tendon and the medial side of the tubercle of scaphoid.

### 4.3. Application in Pulse Diagnosis

According to our research and previous literature reports [2, 5, 7, 8, 23], we speculated that there might be three cases in the exact Cun position (Figure 3(b)): (1) According to the common way to find Cun position, the exact Cun was located in the position of Cun in traditional Chinese medicine (Figure 3(b1)). At this position, we could touch the pulsation of the superficial palmar branch and the pulsation of part of the radial artery, but the length of the radial artery varied greatly, most of which might be less than one finger wide. In addition, the pulsation of the superficial palmar branch and the pulsation of the radial artery might often be confused. (2) According to the pulsation of the radial artery to find Cun position, the exact Cun was between Cun and Guan in traditional Chinese medicine (Figure 3(b2)). The determination of this position depended on the pulsation of the radial artery. The index finger needed to move and be close to the Guan in traditional Chinese medicine. The pulsation of the radial artery was obvious, and its length was one finger wide. (3) Instead of the position of Cun in traditional Chinese medicine, the exact Cun was located in the position of Guan in traditional Chinese medicine (Figure 3(b3)). The position at the medial portion of the bulge of the prominent bone proximal to the palm should be Cun rather than Guan. Tibetan medicine had used this method to determine the position of the Cun and some scholars had suggested using this method [8]. In this position, we could touch the radial artery pulsation, but it might be different from the radial artery touched in the second case. In summary, in any of the above cases, which were more in line with the traditional Chinese medicine theory, we needed further research.

Because the description of Cun in pulse diagnosis in traditional Chinese medicine was simple, the main contribution of this study was to provide some detailed anatomical descriptions of Cun, which was an important supplement to the content of the pulse diagnosis and helpful for the study, research, and clinical practice of the pulse diagnosis.

### 4.4. Study Limitations

However, this study has several limitations. This work only conducted the study of the Cun, without further investigation of the anatomy of the Guan and Chi. This study did not consider differences among human cadavers in age, sex, and body height. Because the variability in the blood vessels of the wrist is more common, the variation of the superficial palmar branch and the radial artery needs further research. Moreover, further studies are needed to define the association between Cun and the superficial palmar branch/the radial artery.

The above findings suggested that whether the pulse in Cun was the radial artery or the superficial palmar branch depends on the vascular distribution in Cun, the region of finger positioning, and the patient's pulse condition.

## 5. Conclusion

This research showed the Cun was located in a quadrilateral depression, formed by the bulge of the prominent bone proximal to the palm, the radial flexor tendon, the tubercle of scaphoid, and the abductor longus muscle tendon. This region contains the radial artery and the superficial palmar branch. All the dissections and observations contributed to the understanding of the anatomical structures of pulse diagnosis in Cun. Because of the differences in the vascular distribution in Cun, the region of finger positioning, and the patient's pulse condition, it could not be generalized that the blood vessel taking pulse in Cun was the radial artery or the superficial palmar branch of radial artery. The pulse point of the radial artery should be taken in the medial side of the bulge of the prominent bone proximal to the palm and the abductor longus muscle tendon, while the pulse point of the superficial palmar branch should be taken in the lateral side of the radial flexor tendon and the medial side of the tubercle of scaphoid.

## Figures and Tables

**Table 1:** Measurement of the Cun position at wrist (n=32).

	mean ±SD
the length of the superficial palmar branch L1 (mm)	17.3±9.2
the pulp width of the index finger L2 (mm)	14.5±2.3
the ratio (L1/L2)	1.2±0.8
the angle between the radial artery and the superficial palmar branch *α* (°)	23.3±9.9

## Data Availability

The data used to support the findings of this study are available from the corresponding author upon request.
